# Case of Excision of the Upper End of the Femur in an Example of Morbus Coxarius

**Published:** 1846-10

**Authors:** William Fergusson

**Affiliations:** Professor of Surgery in King’s College, London


*Case of Excision of the upper end of the Femur in an example of Morbus Coxarius.* By William Fergusson, Esq., Professor of Surgery in King's College, London.—John Clark, æt. 14, suffered for fifteen months from hip disease, and in February, 1845, was in the last stage of hectic. The head of the femur was displaced on the dorsum ilii, and could be felt by the finger passed into a large sinus connected with the disease. The limb on the affected side was between four and five inches shorter than the other, and much distorted by flexion at the knee and hip. There was no indication of disease of the bones of the pelvis, and the head of the femur seemed the principal cause of suffering.

On the 1st of March, 1845, the author made a longitudinal incision on the hip over the head and neck of the bone, and those parts, with a portion of the shaft, including the trochanters, were removed, the bone being cut across with a common saw. The patient bore the operation well; the previous bad symptoms soon disappeared, and in two months he was able to move about the wards of the hospital on crutches, the wound being nearly closed.

The paper concludes with a short historical sketch of the operation, whereby it is shown that this is the second instance in which it has been successfully performed in this country, having been first proposed by Mr. Charles White, of Manchester, in 1770, and first performed by Mr. Anthony White, of the Westminster Hospital, in 1818. —*London Med. Times.*

